# From load-bearing to load-sharing: contact-driven transitions in locked plate constructs

**DOI:** 10.1007/s00402-026-06494-y

**Published:** 2026-09-04

**Authors:** Marianne Hollensteiner, Martina Schindler, Markus Greinwald, Mischa Mühling, Dirk Baumeister, Peter Augat

**Affiliations:** 1 Institute for Biomechanics, Paracelsus Medical University, Salzburg, Austria; 2 Institute for Biomechanics, Berufsgenossenschaftliche Unfallklinik Murnau, Murnau am Staffelsee, Germany; 3 Institute for Modelling and Simulation of Biomechanical Systems, University of Stuttgart, Stuttgart, Germany

**Keywords:** Locked plate osteosynthesis, Bone contact, Interfragmentary motion, Construct stiffness, Plate strain, Bridge plating, Load-sharing

## Abstract

Locked plate osteosynthesis functions as an internal fixator, yet load-induced plate–bone and fragment–fragment contacts – key transitions from load-bearing to load-sharing – remain poorly characterized. This study quantified their effects on construct stiffness, plate strain, and interfragmentary motion under axial loading. Twenty-seven configurations were tested using epoxy-glass surrogate bone fixed with titanium LCP plates, varying fracture gap (3–9 mm), working length (49–121 mm), and plate–bone distance (0–3 mm). Digital image correlation measured strain and motion. Contact events were identified manually from characteristic transitions in the force–displacement response, supported by corresponding changes in plate-surface strain progression. Geometric parameters dominated initial stiffness (125–442 N/mm), decreasing with plate elevation and working length in elevated setups. Within the investigated load range up to 600 N, 16/27 configurations showed non-linear transitions associated with contact events: fragment–fragment contact caused pronounced stiffness increases and strain stagnation at the fracture gap, whereas plate–bone contact was associated with local plate-surface strain stagnation at the location overlying the contact while strain at the fracture gap continued to increase. Interfragmentary motion stagnated after contact, confirming contact-dependent shifts in load transfer. Response surface regression explained 81.9% of the variance in initial stiffness and 89.6% of the variance in axial interfragmentary motion, identifying outcome-specific associations with geometric parameters and selected interaction terms. In conclusion, load-dependent contact events were associated with distinct transitions in construct stiffness, plate-surface strain progression, and interfragmentary motion, complementing the observed associations between geometric parameters and initial mechanical behavior.

## Introduction

Locked plate osteosynthesis has become a cornerstone in the treatment of complex fractures, particularly in osteoporotic bone and metaphyseal regions, due to its angular stability and reduced dependence on plate–bone compression [1–3]. By functioning as an internal fixator, locked plates are designed to bridge fracture gaps while preserving fracture biology and enabling controlled interfragmentary motion [4, 5]. In classical fracture fixation concepts, a distinction is made between load-bearing and load-sharing constructs. Load-bearing implants carry most of the applied load, whereas load-sharing constructs distribute load between implant and bone, thereby allowing controlled deformation at the fracture site. Locked plates configured as internal fixators in bridging constructs function predominantly as load-bearing devices. Under weight-bearing the resulting mechanical forces induce plate deformation and reduction of the interfragmentary gap. In constructs with small interfragmentary gaps this process may ultimately facilitate fracture gap closure and gradual load transfer from the implant to the bone. However, little is known about how load-induced contact between fracture fragments contributes to such transitions in load transfer at different stages of loading [6–8].

Biomechanical investigations of locked plating systems have traditionally focused on construct design parameters such as fracture gap size, working length, and plate–bone distance, as these factors are known to influence construct stiffness, plate strain, and interfragmentary motion [3–5]. Increasing working length and plate–bone distance have generally been associated with reduced construct stiffness and increased construct flexibility, whereas smaller fracture gaps tend to promote higher stiffness and earlier load sharing [3, 9]. These parameters therefore form the basis of most experimental and clinical optimization strategies for locked plate fixation.

However, despite extensive investigation, the reported mechanical effects of these design parameters are not uniform across studies. Contradictory findings have been described particularly for working length, with both stiffness-reducing and stiffness-preserving effects depending on construct configuration and loading conditions [10–13]. These inconsistencies suggest that the mechanical response of locked plate constructs may not be fully captured by linear relationships between geometric parameters and construct behavior.

Similar ambiguities exist for plate–bone distance. While increased plate elevation has been associated with higher plate strain and reduced construct stiffness [14, 15], it is typically treated as a purely geometric parameter. Whether load-induced deformation results in actual plate–bone contact, thereby altering load transfer, is generally not assessed.

Fracture gap size is likewise considered a static parameter, although it directly influences construct stiffness, interfragmentary motion, and mechanical failure risk [3, 16]. The potential for opposing fracture fragments to come into contact under load, and thereby fundamentally change construct behavior, has not been explicitly examined.

Increasing evidence indicates that construct behavior cannot be fully explained by continuous geometric variables alone. Non-linear force–displacement behavior and abrupt stiffness changes during loading have been reported and attributed to additional structural support or contact-related phenomena within the construct [4, 9, 17]. These observations suggest that mechanical response is governed not only by geometric configuration but also by contact conditions developing under load.

In eccentrically plated constructs, loading-induced bending may lead to plate–bone contact on the plated side and fragment–fragment contact on the opposite side, thereby shifting the construct from load-bearing toward load-sharing behavior. Despite their potential relevance, such contact events are rarely systematically assessed, and their occurrence, sequence, and mechanical consequences remain insufficiently characterized.

Understanding these contact-driven mechanisms is therefore essential, as they may substantially influence construct stiffness, strain distribution, and interfragmentary motion, with direct implications for implant loading and fracture healing [18, 19].

The aim of this experimental study was to investigate the role of plate–bone and fragment–fragment contact on the mechanical behavior of locked plate osteosynthesis by varying fracture gap size, working length, and plate–bone distance. Construct stiffness, plate strain, and interfragmentary motion were quantified and related to the occurrence and sequence of different contact states under axial loading, thereby providing a mechanistic framework for understanding load transfer transitions in bridging locked plate constructs.

## Materials and methods

### Experimental setup and specimen preparation

A standardized experimental fracture gap model was used to investigate the influence of fracture gap size, working length, and plate–bone distance on construct stiffness, plate strain, and interfragmentary motion. The bone surrogate segments consisted of cylindrical epoxy–glass fiber composite tubes (Krülit 750, Krüger & Sohn GmbH, Landshut, Germany) with an outer diameter of 30 mm and a wall thickness of 5 mm. A Young’s modulus of 20.47 GPa has previously been reported for the Krülit 750 surrogate material based on axial compression testing [20]. The reported value is comparable to elastic moduli described for human cortical bone [21, 22].

All specimens were stabilized using a titanium locking compression plate (LCP 4.5/5.0 broad, 12 holes, length 224 mm; DePuy Synthes, Oberdorf, Switzerland). Locking screws (5.0 mm diameter, 44 mm length) were inserted with a standardized torque of 4 Nm. Screw placement followed a symmetric configuration around the fracture gap, with fixation starting from the outermost screws and progressing toward the center. To ensure reproducible positioning of the plate relative to the bone surrogate, a custom alignment jig with adjustable fracture gap size and plate–bone distance was used during plate fixation. This jig allowed precise and consistent adjustment of both parameters prior to screw insertion, thereby improving reproducibility and comparability between configurations.

Fracture gap sizes (FG) of 3 mm, 6 mm, and 9 mm were investigated, defined as the axial distance between two parallel fracture fragments. Working length (WL) was defined as the distance between the innermost screws adjacent to the fracture gap and was set to 49 mm, 85 mm, or 121 mm based on the fixed spacing of the plate holes. These different working lengths were achieved by systematic variation of screw placement within the available plate holes, following the approach described by Jitprapaikulsarn et al. [16]. This strategy allowed controlled modification of working length while maintaining a symmetric screw configuration around the fracture gap, ensuring reproducibility and comparability between configurations. Plate–bone distance (PBD) was adjusted to 0 mm, 1 mm, or 3 mm using spacers in the custom-made jig, allowing systematic variation of the lever arm between plate and bone surrogate. In total, 27 distinct configurations were investigated, representing all combinations of three fracture gap sizes, three working lengths, and three plate–bone distances. One separate construct was assembled for each configuration using a new bone-surrogate tube, a new locking plate, and new locking screws.

### Mechanical testing protocol

Biomechanical testing was performed using a servo-electric testing machine (Instron ElectroPuls E3000, Instron, High Wycombe, UK) equipped with a 5 kN load cell. All tests were conducted under axial loading. The proximal and distal ends of the construct were mounted using ceramic ball joints to allow rotational freedom and minimize unintended constraint effects, following established biomechanical testing approaches [23]. Each configuration was subjected to stepwise axial loading starting with a preload of 10 N. Load was increased in increments of 50 N at a rate of 5 N/s until a predefined maximum load of 600 N was reached. This upper limit was selected as a methodological boundary for the quasi-static protocol to investigate configuration-dependent mechanical responses and contact transitions without intentionally progressing to construct failure or high-load testing; it was not intended to reproduce the full range of physiological lower-limb loading. At each load level, the force was held constant for 5 s. Once 600 N was reached, the load was reduced to 10 N within 10 s. Following unloading, the constructs returned to their initial position without observable permanent deformation. Each of the 27 independently assembled constructs underwent three consecutive measurement runs using the same construct. These runs were technical repeated measurements and were not treated as independent experimental replicates.

### Construct stiffness analysis

Axial construct stiffness was determined from the linear regions of the force–displacement curve. To ensure comparability between configurations and to exclude load-dependent contact effects, initial construct stiffness was calculated within a common displacement interval from the preload reference position to 0.4 mm. This interval preceded the first identified contact transition in all investigated constructs. Linear regression was applied separately to each individual measurement run within this interval, and the slope of the regression line was defined as initial construct stiffness. The force–displacement response within the selected interval showed high linearity, with coefficients of determination ranging from R^2^ = 0.9892 to R^2^ = 0.9996 and a median of R^2^ = 0.9992. All data processing and stiffness calculations were performed using MATLAB (MathWorks, Natick, MA, USA).

### Plate strain measurement

Plate strain was quantified using digital image correlation (DIC) with an optical measurement system (ARAMIS Professional 6 M, Zeiss GOM metrology GmbH, Braunschweig, Germany). Prior to testing, the surface of the locking plate was prepared with a stochastic speckle pattern to enable optical strain measurement. The speckle pattern was applied in two steps, consisting of a white base coating (Dupli-Color Aerosol Art Primer, European Aerosols GmbH, Hassmersheim, Germany) followed by spray application of the black stochastic pattern (Dupli-Color Aerosol Art RAL 9005, European Aerosols GmbH, Hassmersheim, Germany).

Strain data were acquired and processed using Zeiss Inspect 2025 software (Zeiss Correlate, Zeiss GOM metrology GmbH, Braunschweig, Germany). The speckle pattern quality was assessed before and after the first measurement using the integrated pattern quality tool, and only configurations with adequate pattern quality throughout testing were included.

**Fig. 1:**
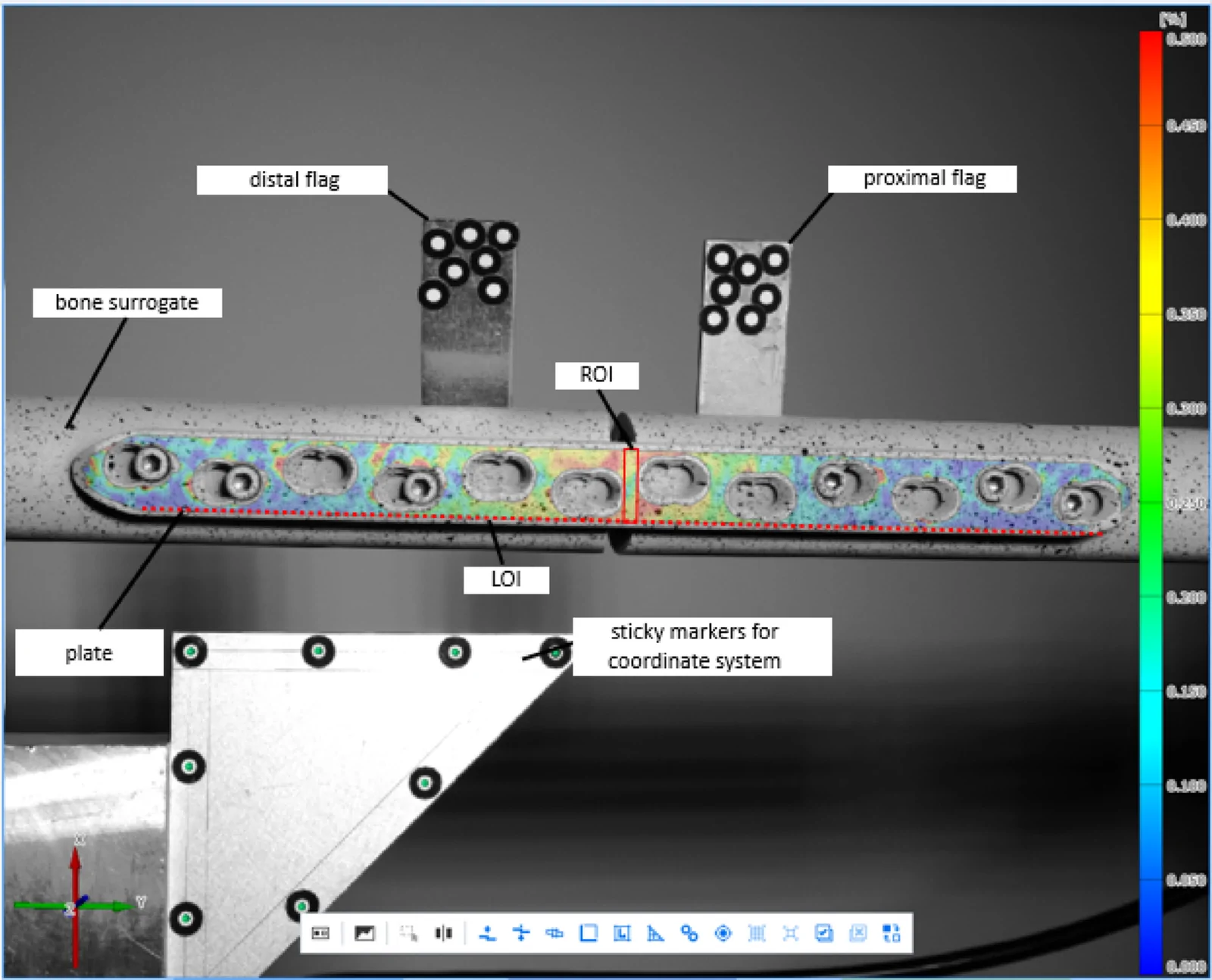
Digital image correlation measurement and evaluation regions. Bone surrogate and locking plate with stochastic speckle pattern for strain analysis. Optical marker flags were used for interfragmentary motion (IFM) measurement. The red box marks the region of interest (ROI) above the fracture gap for maximum principal strain evaluation. The red dotted line represents the longitudinal line of interest (LOI) used for strain distribution analysis. Optical marker flags were attached to screws positioned 10 mm proximal and distal to the fracture gap on the side opposite the plate. Relative displacement was evaluated between the corresponding reference points in the loading direction.

A surface component representing the plate was defined within the software, excluding screw holes and plate edges to avoid artefacts (Fig. 1). The reference state was defined after application of the preload (10 N). Maximum plate strain was evaluated in a defined region of interest (ROI) located centrally above the fracture gap, between the two innermost plate holes adjacent to the gap. Within this region, the maximum principal strain was extracted for each load level, as it captures the dominant tensile deformation independent of plate orientation and bending direction. For longitudinal strain distribution analysis, a continuous line of interest (LOI) was defined on the plate surface lateral to the geometric midline. The midline itself was not used due to alternating screw hole positions that would result in discontinuous strain profiles. The selected section was positioned symmetrically and sufficiently distant from plate edges to avoid boundary artefacts. Strain analysis was limited to a plate length of 160 mm, with the fracture gap center defined at 80 mm. The terminal plate regions were excluded from evaluation.

Strain values were extracted at equidistant points with a spacing of 0.1 mm along the defined section. Data were exported for axial loads between 200 N and 600 N in increments of 50 N and further processed in MATLAB (MathWorks, Natick, MA, USA).

### Interfragmentary motion analysis

To enable robust tracking of relative fragment motion, rigid optical marker flags (Fig. 1) were attached to screws inserted centrally into the proximal and distal surrogate segments on the side opposite the fixation plate. The screws were positioned 10 mm proximal and distal to the fracture gap, respectively. The screw-end positions at the surrogate surface were defined as reference points within the DIC system.

Relative displacement was quantified as the direct relative displacement between the proximal and distal reference points in the loading direction. Because the reference points were located outside the fracture plane, the measured displacement represents the relative motion of the marker locations and may include contributions from both fragment translation and rotation. IFM values were evaluated at the maximum load level of 600 N.

### Identification of contact states

Load-dependent contact events were identified manually from characteristic transitions in the force–displacement response. No predefined numerical threshold for slope change and no automated detection algorithm were applied. Fragment–fragment contact was assigned when the force–displacement curve exhibited a pronounced transition toward a near-vertical response, consistent with direct load transfer between the opposing surrogate bone fragments. Plate–bone contact was assigned to distinct additional transitions in the force–displacement response consistent with a change in construct stiffness.

The mechanical interpretation of these transitions was supported by the corresponding plate-strain progression. Contact-related transitions in the force–displacement response coincided with stagnation or distinct changes in plate-surface strain progression. In configurations exhibiting multiple distinct transitions, sequential contact events were assigned separately based on the sequence of changes in the mechanical and strain responses. Because contact onset was identified manually without a predefined numerical slope threshold, less pronounced transitions may be sensitive to measurement resolution and evaluator interpretation; no formal sensitivity analysis using alternative threshold criteria was performed.

### Statistical analysis

For each of the 27 geometric configurations, three consecutive measurements were obtained from the same construct. These measurements represent technical repeated measurements rather than independent experimental replicates. For statistical analysis, the three repeated measurements were averaged to obtain one value per independently assembled configuration, resulting in 27 independent observations.

To assess associations between the geometric parameters and the mechanical outcomes, second-order response surface regression models were fitted separately for initial construct stiffness and axial interfragmentary motion. Fracture gap size (FG), working length (WL), and plate–bone distance (PBD) were entered as continuous predictors. The models included linear terms for FG, WL, and PBD, quadratic terms for each predictor, and all pairwise two-way interaction terms. A three-way interaction term was not included to avoid overparameterization given the 27 independent configurations. Predictor variables were centered and standardized prior to model fitting. Model fit was assessed using R² and adjusted R². Statistical inference for individual model terms was based on heteroskedasticity-consistent HC3 standard errors and corresponding p-values. Homoscedasticity was assessed using the Breusch–Pagan test. Because this test indicated heteroskedasticity, HC3 robust standard errors were used for statistical inference. The level of significance was set to α = 0.05.

## Results

### Initial construct stiffness

**Fig. 2:**
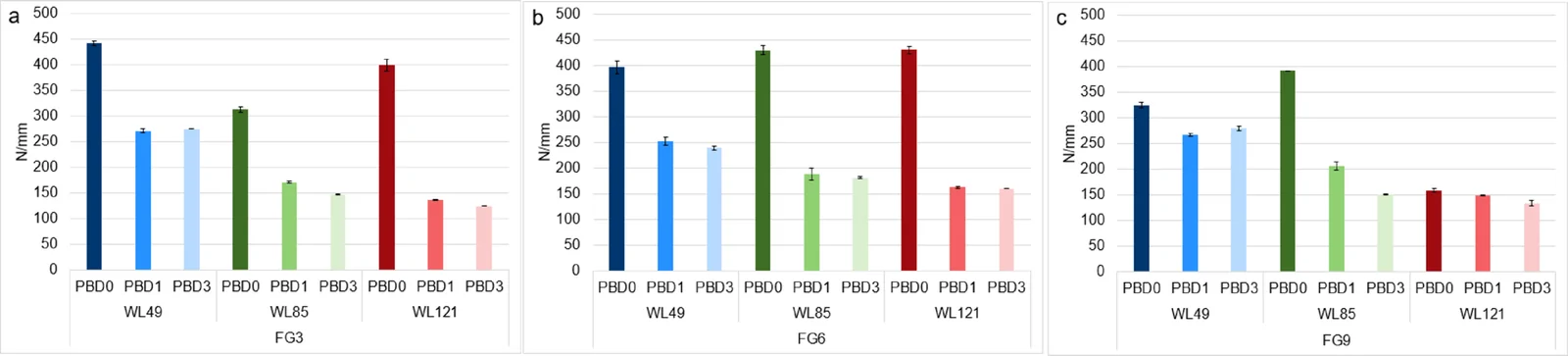
Initial construct stiffness (mean ± SD) determined within the pre-contact load range for all configurations. Data are presented separately for **a** FG3, **b** FG6, and **c** FG9. Within each subplot, working length (WL49–WL121) is color-coded (blue tones: WL49; green tones: WL85; red tones: WL121). Increasing plate–bone distance (PBD0, PBD1, PBD3) is represented by progressively lighter shades within each working length group. Error bars indicate the standard deviation of three consecutive technical repeated measurements performed on the same construct

Initial construct stiffness ranged from 125.4 ± 0.2 N/mm to 441.8 ± 4.3 N/mm across the 27 investigated configurations (Fig. 2). The second-order response surface regression model explained 81.9% of the variance in initial construct stiffness (R² = 0.819; adjusted R² = 0.723). Using HC3 robust standard errors, significant associations were observed for WL (*p* = 0.017), PBD (*p* < 0.001), and the quadratic PBD term (*p* = 0.002), whereas no significant linear or quadratic association with FG and no significant pairwise interaction terms were identified. Across all FG sizes and WL, constructs with direct plate–bone contact (PBD0) consistently exhibited higher initial stiffness than constructs with elevated plates (PBD1 and PBD3). For each combination of FG and WL, the transition from PBD0 to PBD1 was associated with a marked reduction in stiffness. In contrast, the difference between PBD1 and PBD3 was comparatively small. This pattern was observed across all fracture gap categories, indicating a consistent effect of plate elevation on initial construct stiffness.

The influence of working length depended on plate–bone distance. For configurations with elevated plates (PBD1 and PBD3), increasing working length was associated with a progressive reduction in stiffness across all fracture gap sizes. In contrast, when the plate was positioned directly on the bone surrogate (PBD0), no uniform relationship between working length and stiffness was observed. Depending on fracture gap size, stiffness either decreased, increased, or remained similar across working lengths.

Variation in fracture gap size resulted in differences in initial stiffness; however, no consistent monotonic trend with fracture gap size was observed across working lengths and plate–bone distances.

### Contact occurrence and sequence

**Fig. 3:**
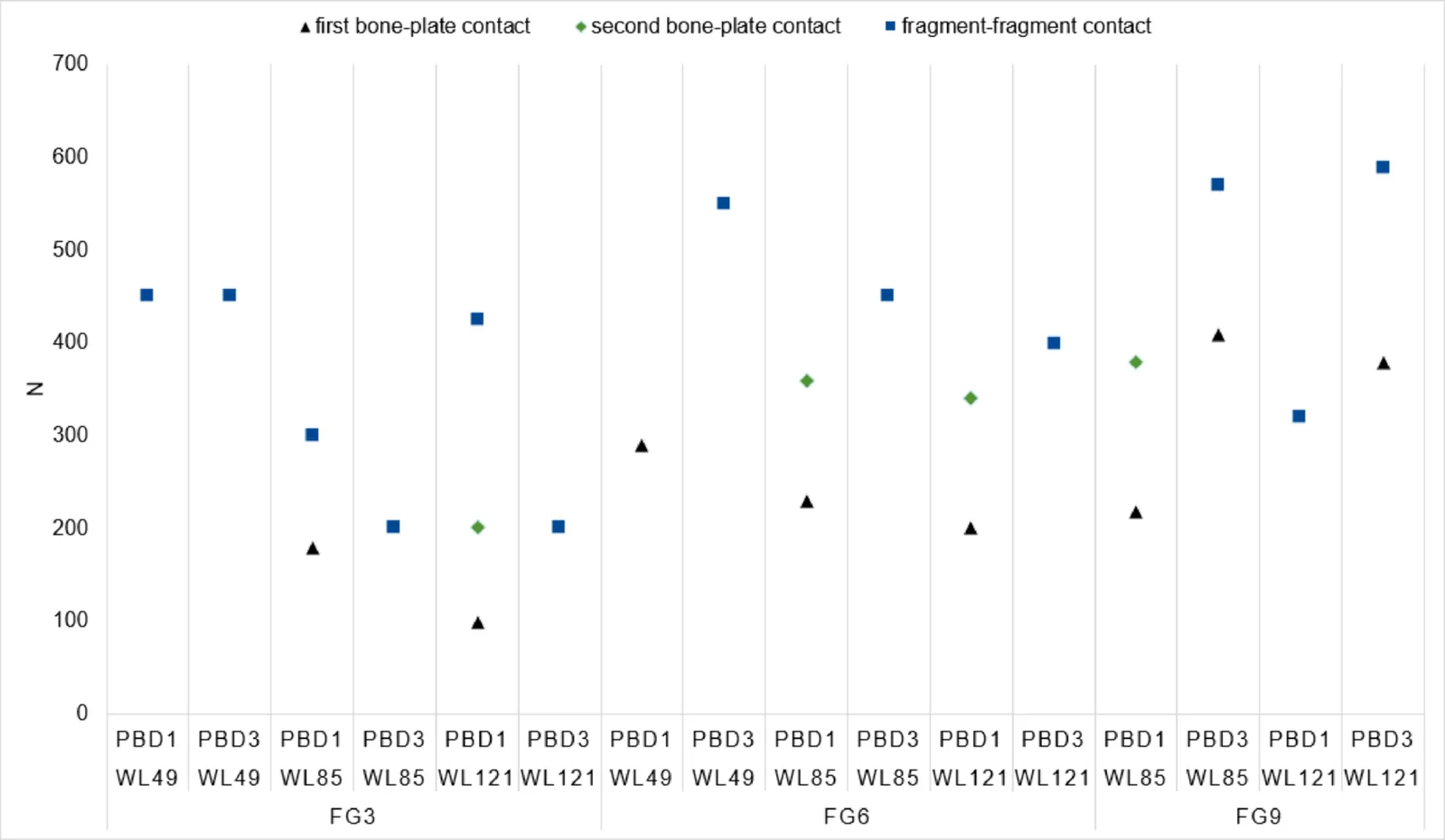
Contact onset loads for each configuration, grouped by fracture gap size and ordered by working length and plate–bone distance. Squares indicate fragment–fragment contact, triangles first bone–plate contact, diamonds second bone–plate contact. Multiple symbols per configuration represent sequential contacts

Load-dependent contact events were observed in 16 of the 27 configurations (Fig. 3). Accordingly, configurations without detected contact should be interpreted as showing no contact up to 600 N rather than as intrinsically contact-free. Contacts occurred either as isolated fragment–fragment contact, isolated bone–plate contact, or as sequential contact events involving two or, in individual configurations, three distinct contact transitions within the same construct. The load at first contact ranged from 80 N to 590 N. The earliest contact was observed at 80 N (FG3–WL121–PBD1), whereas the latest last-contact events occurred at 570 N and 590 N in elevated-plate configurations (FG9–WL85–PBD3; FG9–WL121–PBD3).

Contacts were observed exclusively in constructs with elevated plates (PBD1 or PBD3). Fragment-fragment contact occurred across all fracture gap sizes and working lengths and was frequently observed as the sole contact type in a configuration.

Sequential contact events were exclusively observed in constructs with PBD1, where both fragment–fragment followed by bone–plate contact and two successive bone–plate contacts occurred. In contrast, in PBD3 configurations, sequential contacts were limited to fragment–fragment contact following an initial bone–plate contact, and no second bone–plate contact was observed. Such multiple contact transitions were more frequently observed at WL85 and WL121 than at WL49. No uniform monotonic relationship between fracture gap size and contact load was observed. Contact events occurred across all fracture gap groups (FG3, FG6, FG9), with a broad distribution of onset loads within each category.

### Contact-induced stiffness transitions

Force–displacement curves exhibited non-linear behavior with distinct slope changes during progressive loading. All configurations showed an initial linear regime within the predefined displacement interval used to calculate initial construct stiffness. Beyond this range, several constructs demonstrated a gradual reduction in slope prior to contact formation, consistent with plate bending-dominated behavior. Upon onset of plate–bone contact, the force–displacement response changed, with a partial increase in slope compared to the immediately preceding regime. In most configurations, however, this contact-dependent stiffness remained lower than the initial construct stiffness.

**Fig. 4:**
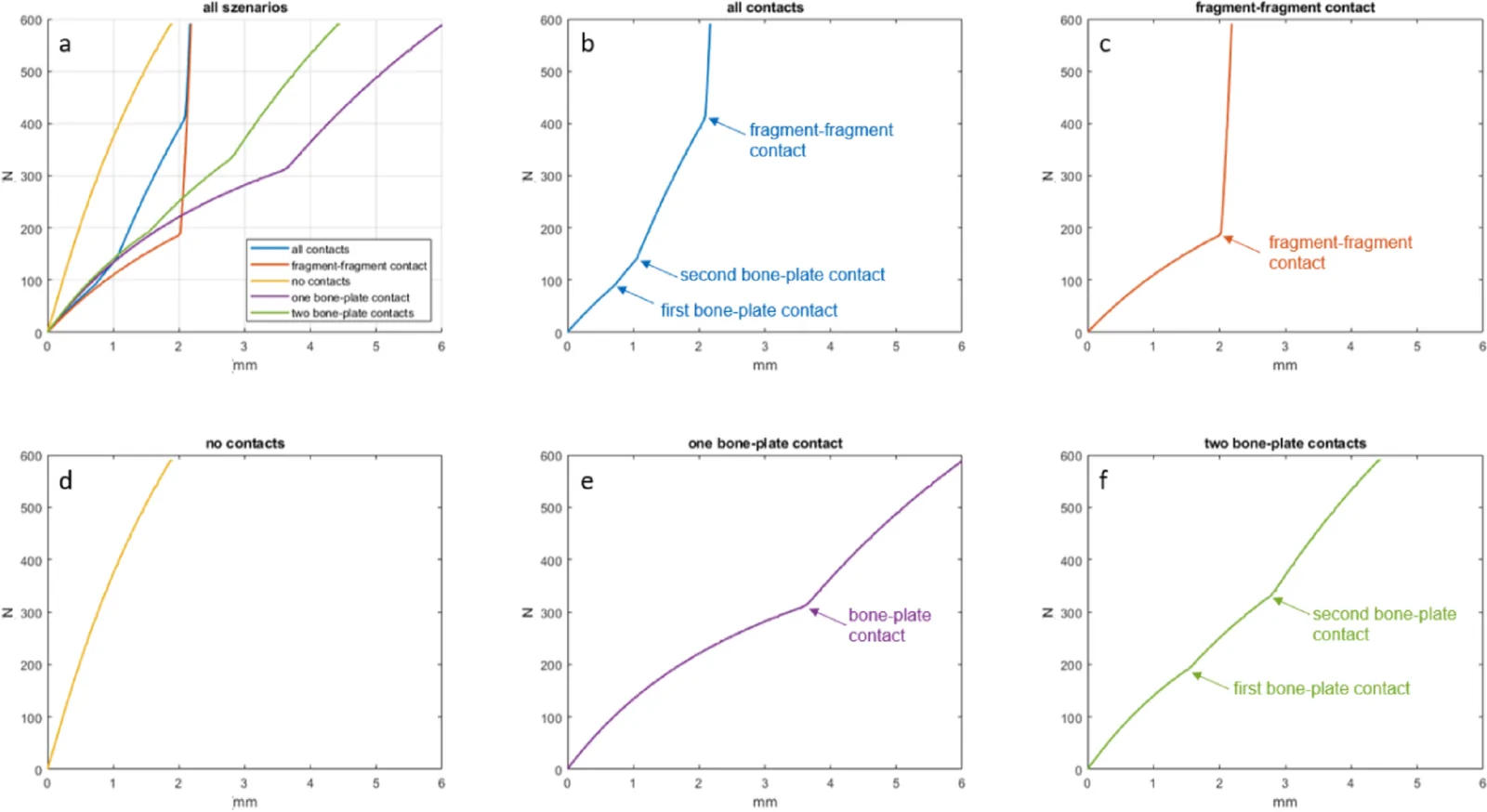
Force–displacement curves illustrating different contact scenarios. **a** Overview of all scenarios plotted simultaneously. **b** Configuration exhibiting three sequential contact events (two plate–bone contacts followed by one fragment–fragment contact, FG3-WL121-PBD1). **c** Configuration with fragment–fragment contact (FG3-WL121-PBD3). **d** Configuration without contact (FG6-WL121-PBD0). **e** Configuration with one plate–bone contact (FG9-WL85-PBD1). **f** Configuration with two plate–bone contacts (FG6-WL121-PBD1). Arrows indicate contact events

Fragment–fragment contact was associated with a more pronounced increase in slope compared to plate–bone contact, reflecting a substantial transition in construct behavior. In configurations with sequential contact events, multiple slope transitions were observed within a single loading sequence, reflecting successive changes in structural support conditions (Fig. 4).

### Interfragmentary motion

**Fig. 5:**
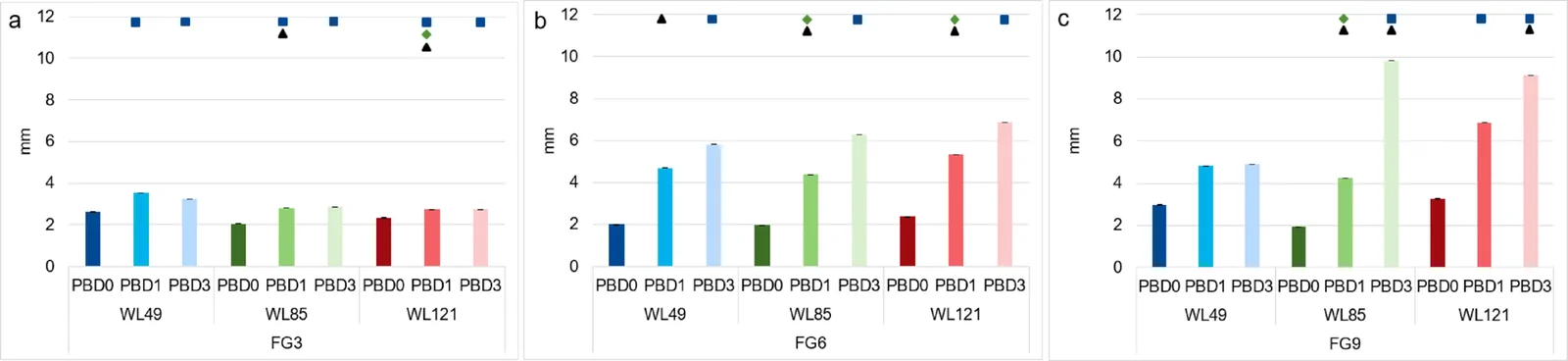
Mean axial interfragmentary motion (IFM) ± SD at 600 N is shown for all fracture gap sizes (FG), working lengths (WL), and plate–bone distances (PBD). Squares indicate fragment–fragment contact, triangles first bone–plate contact, diamonds second bone–plate contact. Multiple symbols per configuration represent sequential contacts. Error bars indicate the standard deviation of three consecutive technical repeated measurements performed on the same construct

Axial interfragmentary motion (IFM), determined at a maximum axial load of 600 N, ranged from 1.9 mm to 9.8 mm depending on the specific configuration (Fig. 5). The smallest IFM was observed in FG9–WL85–PBD0, whereas the largest IFM occurred in FG9–WL85–PBD3. For the configuration exhibiting the largest IFM (FG9–WL85–PBD3), the corresponding actuator displacement at 600 N was 8.69 mm, compared with a marker-based relative axial displacement of 9.82 mm.

The second-order response surface regression model explained 89.6% of the variance in axial IFM (R² = 0.896; adjusted R² = 0.840). Using HC3 robust standard errors, significant associations were observed for FG (*p* = 0.0016), PBD (*p* < 0.001), the quadratic PBD term (*p* = 0.027), and the FG × PBD interaction (*p* = 0.039). The linear association with WL and the remaining pairwise interaction terms did not reach statistical significance.

Across all investigated FG and WL, constructs with direct plate–bone contact (PBD0) consistently exhibited the lowest IFMs values. However, the relative ordering between PBD1 and PBD3 was not uniform across all FG/WL combinations.

WL influenced axial IFM within each FG and PBD category. For most configurations, increasing WL was associated with increased absolute displacement; however, the magnitude and direction of this relationship varied depending on the selected FG and PBD.

FG further modulated axial IFM. Larger fracture gaps were associated with higher displacement magnitudes in most geometric combinations, although this relationship was not strictly monotonic across all configurations.

Evaluation of the displacement progression under increasing load demonstrated that alterations in IFM coincided with mechanically identified contact events. In several configurations, the onset of fragment–fragment or bone–plate contact corresponded to a reduction or partial stagnation of displacement progression beyond the respective contact load, indicating a transition in the mechanical response following contact formation.

### Maximum plate strain at fracture gap

**Fig. 6:**
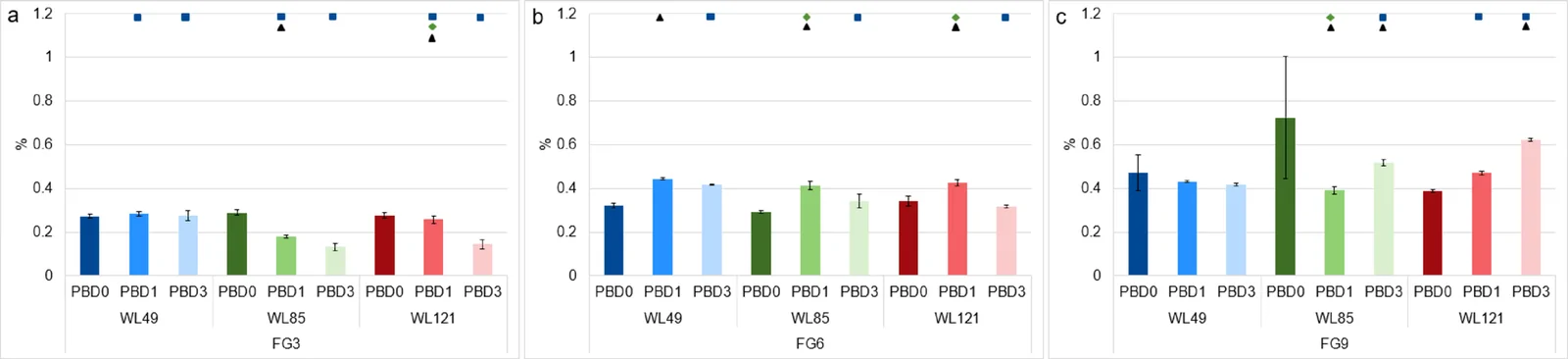
Mean maximum principal strain measured in the central plate region directly above the fracture gap is shown for all fracture gap sizes (FG), working lengths (WL), and plate–bone distances (PBD). Squares indicate fragment–fragment contact, triangles first bone–plate contact, diamonds second bone–plate contact. Multiple symbols per configuration represent sequential contacts. Error bars indicate the standard deviation of three consecutive technical repeated measurements performed on the same construct

Maximum principal strain in the central plate region directly above the fracture gap increased predominantly with fracture gap size (Fig. 6). At 600 N, strain values ranged approximately from 0.13 to 0.29% in FG3, 0.29–0.44% in FG6, and 0.39–0.72% in FG9, indicating a clear geometric effect of FG on plate deformation.

Within identical FG groups, no consistent monotonic relationship was observed for WL or PBD. Strain magnitudes varied across configurations, and no uniform pattern could be identified between constructs without detected contact up to 600 N and constructs exhibiting contact within the investigated load range across all fracture gap sizes. In smaller fracture gaps, configurations without detected contact up to 600 N did not consistently exhibit higher strain values.

Fragment–fragment contact was associated with reduced strain magnitudes in several configurations; however, terminal strain levels at 600 N were not uniformly determined by contact state alone. Plate–bone contact without fragment closure did not consistently reduce strain compared to contact-free constructs.

### Contact-associated strain transitions

**Fig. 7:**
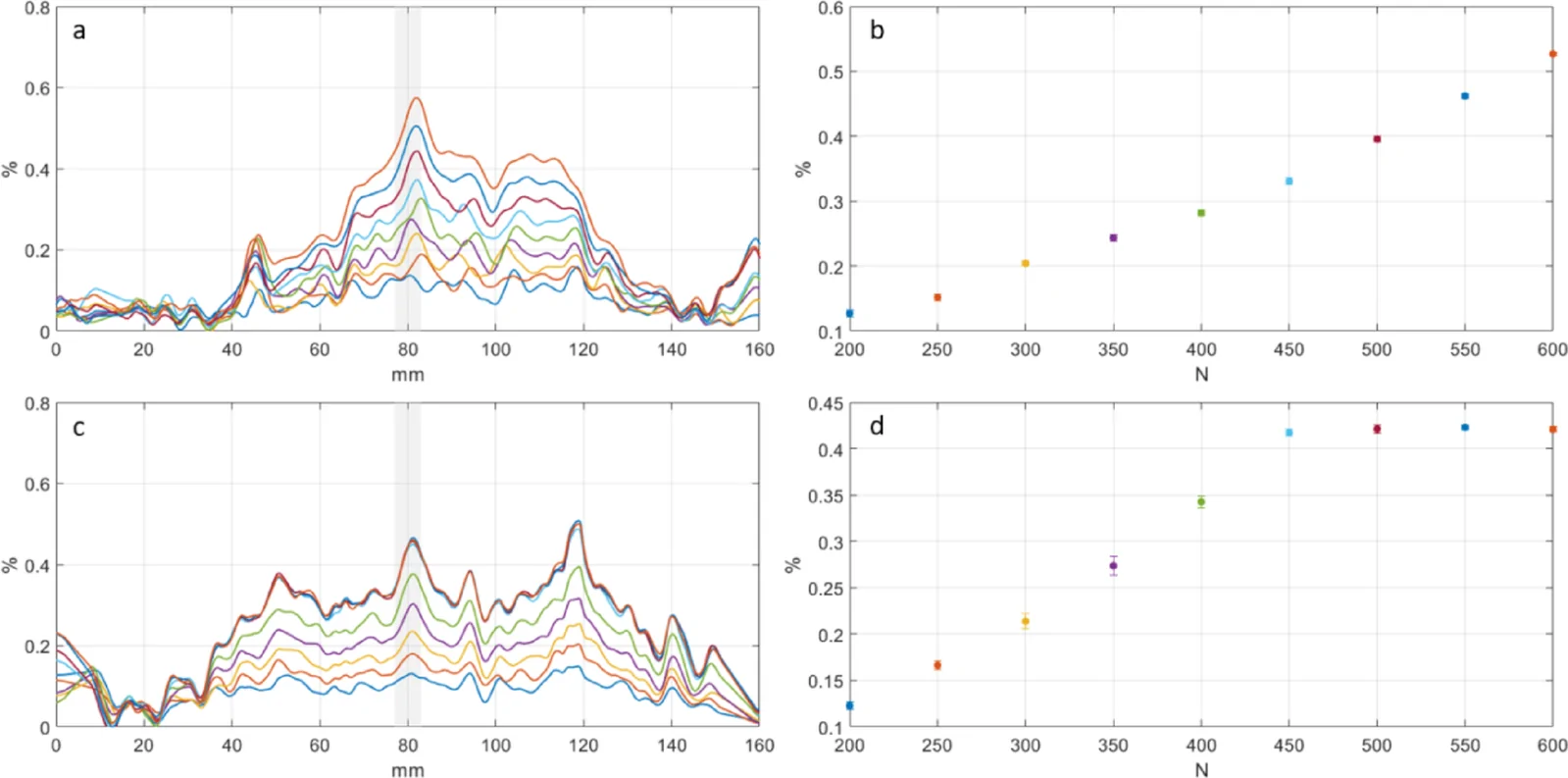
Strain distribution along the plate (**a**, **c**) and corresponding load–strain relationship at the fracture gap (**b**, **d**) for a representative contact-free configuration (**a**, **b**) and a representative configuration with fragment–fragment contact (**c**, **d**). Gray-shaded area indicates the fracture gap. Strain profiles are shown for load levels from 200 to 600 N in 50-N increments. In the contact-free configuration, strain increases proportionally with load, resulting in approximately equally spaced strain curves and a linear load–strain relationship. After fragment–fragment contact onset (~ 400 N), strain at the fracture gap reaches a plateau despite further load increase

To illustrate the effect of contact formation on strain evolution, one representative configuration without detected contact up to 600 N and one representative configuration with fragment–fragment contact were analyzed over the load range from 200 to 600 N in 50-N increments (Fig. 7). The left panels display the strain distributions along the plate for each load level, whereas the right panels show the corresponding load–strain relationship in the evaluated region. In the configuration without detected contact up to 600 N (Fig. 7a, b), the strain curves remained approximately equally spaced over the entire loading range, indicating a proportional increase in strain with load. This was reflected by an approximately linear load–strain relationship at the fracture gap.

In the configuration exhibiting fragment–fragment contact (Fig. 7c, d), strain initially increased in a similar manner up to contact onset at approximately 400 N. Beyond this point, the strain curves converged and strain at the fracture gap no longer increased substantially despite further load increase, resulting in a plateau in the load–strain relationship.

In configurations with plate–bone contact, local plate-surface strain at the location overlying the contact region stagnated after contact onset, whereas plate-surface strain above the fracture gap continued to increase with load. This pattern indicates a local change in plate deformation associated with plate–bone contact, without the strain stagnation above the fracture gap observed after fragment–fragment contact.

## Discussion

The aim of this study was to investigate the role of plate–bone and fragment–fragment contact in locked plate osteosynthesis by varying fracture gap size, working length, and plate–bone distance and relating these parameters to construct stiffness, plate strain, and interfragmentary motion under axial loading. The results demonstrate that geometric design parameters were associated with initial construct stiffness and interfragmentary motion, while load-dependent contact events represented distinct mechanical transitions associated with changes in construct stiffness, strain distribution, and interfragmentary motion.

Consistent with previous biomechanical investigations, working length and plate–bone distance were significantly associated with initial construct stiffness, whereas fracture gap size showed no significant independent association in the response surface model [3, 5, 14]. The absence of a significant association between fracture gap size and initial construct stiffness differs from previous reports describing higher construct stiffness with smaller fracture gaps. This discrepancy may partly reflect the definition of initial stiffness used in the present study. Stiffness was deliberately determined within a common pre-contact displacement interval of 0–0.4 mm, thereby characterizing the initial mechanical response before load-dependent fragment–fragment contact occurred. Consequently, fracture gap size may become mechanically more relevant at larger displacements as the fragments approach contact, whereas its association with stiffness within the initial pre-contact regime was limited in the present model. Elevated plates exhibited reduced initial stiffness compared to constructs with direct plate–bone contact, and increasing working length reduced stiffness in elevated constructs [11, 12, 15]. However, no uniform monotonic relationship between working length and stiffness was observed in constructs with direct plate–bone contact, indicating interaction effects between geometric parameters.

Beyond the initial linear regime, several configurations demonstrated non-linear force–displacement behavior with distinct slope transitions. These slope changes coincided with mechanically identified plate–bone or fragment–fragment contact events. Similar non-linear behavior has been reported in locking constructs and attributed to changes in load transfer mechanisms [4, 9]. In the present study, these stiffness transitions were associated with the corresponding contact states.

Fragment–fragment contact was associated with a pronounced increase in construct stiffness compared to plate–bone contact and was consistently accompanied by overlapping strain curves beyond the contact load. This indicates that further load increments did not result in additional plate strain once fragment contact was established. In contrast, plate–bone contact was associated with local stagnation of plate-surface strain at the location overlying the contact region, whereas plate-surface strain above the fracture gap continued to increase with load, consistent with a local redistribution rather than an arrest of plate deformation.

These changes indicate a modification of the bending profile of the construct once local support from the underlying bone was established. Although working length was defined geometrically as the distance between the innermost screws adjacent to the fracture gap, load-induced plate–bone contact alters the mechanically active span of the plate. Once plate–bone contact occurs, the plate is locally supported, reducing the unsupported bending segment and thereby modifying the effective working length under load. Configurations with two sequential plate–bone contacts exhibited increasingly localized constraint effects, consistent with progressive shortening of the unsupported bending region. This dynamic modification of effective working length provides a mechanical explanation for the non-monotonic stiffness relationships observed in constructs with direct plate–bone contact and may partly account for the contradictory working length effects reported in previous biomechanical studies [10–13, 24].

Strain distribution analysis further demonstrated that contact formation altered the mechanical response of the construct in a contact-specific manner. In contact-free configurations, strain increased proportionally with load, consistent with approximately linear load–strain behavior at the fracture gap. After fragment–fragment contact onset, strain at the fracture gap stagnated despite further load increase, indicating a transition toward direct load transfer between the fragments. In contrast, plate–bone contact was associated with local stagnation of plate-surface strain at the location overlying the contact region, whereas plate-surface strain above the fracture gap continued to increase with load. This finding suggests that fragment–fragment contact and plate–bone contact represent mechanically distinct forms of load redistribution.

Interfragmentary motion showed corresponding changes at contact onset. In several configurations, contact formation coincided with partial reduction or stagnation of displacement progression under increasing load. Thus, contact state influenced not only global construct stiffness but also local strain distribution and fragment motion.

Several limitations should be considered. First, the experiments were conducted using standardized epoxy–glass composite bone surrogates. While this ensured high reproducibility and controlled mechanical properties, it does not replicate the heterogeneity and anisotropy of human bone tissue [21, 22]. Several studies cited to contextualize general mechanical principles were conducted in veterinary or preclinical experimental models. While these studies provide relevant biomechanical insights, species- and model-specific differences limit direct extrapolation to human fracture fixation. In addition, one independently assembled construct was tested for each geometric configuration, with three consecutive measurements performed on the same construct. These repeated measurements quantify technical repeatability but do not constitute independent replication within individual factorial cells. Statistical modeling was therefore performed on one averaged value per independently assembled configuration. Furthermore, contact state was not implemented as an independently controlled experimental factor and was not subjected to direct causal effect estimation. Contact-related changes should therefore be interpreted as descriptive within-construct mechanical observations, and the present study cannot separate an independent effect of contact from the geometric conditions under which contact developed. Furthermore, interfragmentary motion was quantified using reference points located 10 mm proximal and distal to the fracture gap rather than directly within the fracture plane. Consequently, the reported values represent relative displacement at the marker locations and may include rotational components in addition to pure fracture-gap closure.

Second, loading was limited to quasi-static axial compression up to 600 N. Despite the large displacements observed in highly compliant configurations, constructs returned to their initial position following unloading without observable permanent deformation, supporting predominantly elastic behavior within the investigated loading range. Under physiological conditions, locked plate constructs are exposed to multidirectional and time-varying loads, including torsion, bending, and combined loading. Such loading modes may alter relative fragment motion and plate deformation and may therefore substantially affect the load level, location, sequence, and type of plate– bone and fragment–fragment contact events. Moreover, contact behavior is likely to depend on fracture morphology. Previous experimental work demonstrated that realistic, interlocking distal femur fracture geometries exhibit distinct mechanical behavior compared with standardized planar osteotomies, particularly under torsional and combined loading conditions [25]. Consequently, the contact patterns and transition sequences observed in the present study should be considered specific to both the applied axial loading condition and the standardized planar fracture geometry and should not be assumed to persist under multidirectional loading or more complex fracture morphologies. In addition, the upper load limit of 600 N restricts conclusions regarding contact occurrence to the investigated load range. Configurations without detected contact may develop plate–bone or fragment–fragment contact at higher loads, particularly because contact transitions were observed close to the upper test limit. Third, contact onset was identified manually from characteristic transitions in the force–displacement response without a predefined numerical slope threshold or automated detection algorithm. Although the mechanical interpretation was supported by corresponding changes in plate-strain progression, the exact contact-onset load may depend on measurement resolution and evaluator interpretation. Contact loads should therefore be interpreted as experimentally identified transition points rather than as threshold values with absolute precision.

The present findings can also be interpreted in the context of the classical distinction between load-bearing and load-sharing fixation constructs. In the pre-contact regime, the locked plate essentially behaves as a load-bearing device, carrying the majority of the applied load and limiting interfragmentary motion. Once fragment–fragment contact is established, the construct transitions toward a more load-sharing state, in which the bone fragments contribute substantially to load transfer and plate strain stagnates despite further load increases. Plate–bone contact represents an intermediate situation in which load is partially redistributed along the plate and toward the bone surface, without complete arrest of plate deformation. These results suggest that contact formation under load acts as a discrete mechanical transition between predominantly load-bearing and increasingly load-sharing behavior in locked plating constructs, rather than a gradual, purely geometry-driven change [6–8].

The present findings have implications primarily for the design and interpretation of biomechanical studies of bridging locked plate constructs. Mechanical responses obtained at a given load level may reflect different load-transfer states depending on whether contact transitions have occurred during loading. Explicit consideration and reporting of such transitions may therefore improve the interpretation and comparability of construct stiffness, plate-strain, and fragment-motion measurements across experimental configurations. Beyond biomechanical testing, the potential relevance of load-dependent contact events to fracture healing remains hypothetical, particularly because the mechanobiological interpretation of interfragmentary motion and strain depends on the local mechanical environment rather than on a single displacement or strain threshold [26]. Validation under physiologically representative loading conditions, realistic fracture morphologies, biological models, and ultimately clinical investigation is therefore required.

## Conclusion

In summary, this study demonstrates that the mechanical behavior of locked plate osteosynthesis is not fully determined by geometric design parameters alone. While construct stiffness, plate strain, and interfragmentary motion varied across combinations of fracture gap size, working length, and plate–bone distance, load-dependent contact events were associated with abrupt transitions in mechanical responses.

Fragment–fragment contact resulted in strain stagnation at the fracture gap, indicating a transition toward load-sharing fixation. In contrast, plate–bone contact was associated with local stagnation of plate-surface strain at the location overlying the contact region, while plate-surface strain above the fracture gap continued to increase with load. These contact-dependent transitions manifested consistently across force–displacement behavior, interfragmentary motion, and strain distribution patterns.

Taken together, these findings indicate that load-dependent contact events were associated with distinct changes in load-transfer behavior in the tested bridging constructs. These descriptive observations complement the configuration-level associations identified for geometric parameters but do not establish contact state as an independent causal factor. Load-induced contact events should therefore be considered when interpreting construct stiffness and plate-strain responses in biomechanical tests, while their independent contribution and clinical relevance require further investigation in specifically designed studies.

## Data Availability

The datasets generated and analyzed during the current study are not publicly available but are available from the corresponding author upon reasonable request.
